# Primary infection by *Pneumocystis* induces Notch-independent Clara cell mucin production in rat distal airways

**DOI:** 10.1371/journal.pone.0217684

**Published:** 2019-06-06

**Authors:** Andrea Méndez, Diego A. Rojas, Carolina A. Ponce, Rebeca Bustamante, Caroll J. Beltrán, Jorge Toledo, Victor A. García-Angulo, Mauricio Henriquez, Sergio L. Vargas

**Affiliations:** 1 Programa de Microbiología y Micología, Instituto de Ciencias Biomédicas (ICBM), Facultad de Medicina Universidad de Chile, Santiago, Chile; 2 Servicio de Gastroenterología, Hospital Clínico Universidad de Chile y Facultad de Medicina, Universidad de Chile, Santiago, Chile; 3 Laboratorio de Análisis Imágenes Científicas, SCIAN-lab, Instituto de Neurociencias Biomédicas (BNI), Facultad de Medicina Universidad de Chile, Santiago, Chile; 4 Programa de Fisiología y Biofísica, Instituto de Ciencias Biomédicas (ICBM), Facultad de Medicina Universidad de Chile, Santiago, Chile; University of Pittsburgh, UNITED STATES

## Abstract

Clara cells are the main airway secretory cells able to regenerate epithelium in the distal airways through transdifferentiating into goblet cells, a process under negative regulation of the Notch pathway. *Pneumocystis* is a highly prevalent fungus in humans occurring between 2 and 5 months of age, a period when airways are still developing and respiratory morbidity typically increases. *Pneumocystis* induces mucus hyperproduction in immunocompetent host airways and whether it can stimulate Clara cells is unknown. Markers of Clara cell secretion and Notch1 activation were investigated in lungs of immunocompetent rats at 40, 60, and 80 days of age during *Pneumocystis* primary infection with and without Valproic acid (VPA), a Notch inducer. The proportion of rats expressing mucin increased in *Pneumocystis*-infected rats respect to controls at 60 and 80 days of age. Frequency of distal airways Clara cells was maintained while mRNA levels for the mucin-encoding genes *Muc5B* and *Muc5ac* in lung homogenates increased 1.9 and 3.9 times at 60 days of infection (*P*. *=* 0.1609 and *P*. *=* 0.0001, respectively) and protein levels of the Clara cell marker CC10 decreased in the *Pneumocystis*-infected rats at 60 and 80 days of age (*P*. *=* 0.0118 & P. = 0.0388). CC10 and Muc5b co-localized in distal airway epithelium of *Pneumocystis*-infected rats at day 60. Co-localization of Muc5b and Ki67 as marker of mitosis in distal airways was not observed suggesting that Muc5b production by Clara cells was independent of mitosis. Notch levels remained similar and no transnucleation of activated Notch associated to *Pneumocystis* infection was detected. Unexpectedly, mucus was greatly increased at day 80 in *Pneumocystis*-infected rats receiving VPA suggesting that a Notch-independent mechanism was triggered. Overall, data suggests a Clara to goblet cell transdifferentiation mechanism induced by *Pneumocystis* and independent of Notch.

## Introduction

Clara or Club cells are a group of epithelial cells in the airway which secrete Clara Cell Secretory Protein (CCSP or CC10)[1]. They are the most abundant cells in the airway of rodents (57%)[2] and their proportion may vary among different species. In humans, Clara cells represent 22% of epithelial cells in distal airway, the location where they are more abundant[3]. Clara cells have functions in immune response, metabolism of toxic substances and epithelial regeneration[4–6]. Moreover, these cells are considered the major Transit Amplifying (TA) cell population in the airway epithelium, which regenerate epithelial cells in normal lungs as has been documented after a lung injury in mice[4].

Unlike Clara cells, goblet cells are scarce in normal airways, representing 11% of total epithelial cells in humans. In rodents, they comprise less than 5% in the proximal airway, while nearly absent in the distal airway[2, 3]. Antigenic stimuli can induce an increase in goblet cells in proximal and distal airways, through a mitosis-independent mechanism[2, 7, 8]. Studies have shown colocalization of goblet cell markers with CCSP in models of asthma induced by ovalbumin and *Aspergillus fumigatus* [2, 7, 8], and subsequent studies in postnatal development have demonstrated full transdifferentiation from Clara to goblet cells in the airway[9].

Differentiation from Clara to goblet cells in postnatal development is negatively regulated by the Notch signaling pathway [9]. The role of Notch pathway in regulating transformation of Clara to mucous cells is well established[10], and can be reversed by using Notch antagonists that induce an increase in goblet cells in human epithelial cells[11]. Notch is a master regulatory circuit involved in cellular proliferation, differentiation and apoptosis[12]. The Notch intracellular domain (NICD) arising from Notch cleavage translocates into the nucleus, where it interacts with CSL, a DNA binding transcriptional regulator. NICD-CSL complex activates the transcription of various downstream effectors, among which are the Hes/Hey group of effectors [9, 13]. Studies in lung development have shown a reduction of Clara cells in mice with suppression of Notch or with a deletion in the Hes1 gene, a Notch effector[9, 14, 15]. Furthermore, it has been shown that suppression of the Notch pathway induces transdifferentiation from Clara to goblet cells in proximal airways during postnatal development[12]. Accordingly, the Notch pathway regulates the transcription of genes related to goblet cell phenotype, such as the gene coding for Muc5ac, a main secreted gel-forming mucin, which is repressed by the Notch effectors Hes1 and Hes5[12, 16].

Transdifferentiation from Clara to goblet cells has also been documented in rodent models of asthma[2, 7, 8]. In addition, an increase in goblet cells with reduction in Clara cells expressing CCSP that switch to coexpress CCSP and Muc5ac, has also been described in rodents during infection by Sendai Virus or Respiratory Syncytial Virus (RSV)[17, 18]. In addition, reduced expression of the Notch receptor and its effector proteins Hes/Hey has been found in the airway epithelium of patients with Chronic Obstructive Pulmonary Disease (COPD)[19].

*Pneumocystis* is a highly prevalent fungus in immunocompetent infants who acquire the primary infection before 6 months of age and in adults who can carry small burdens of *Pneumocystis* organisms[20, 21]. *Pneumocystis* infection in immunocompetent infants is associated to increased levels of the MUC5AC and MUC5B mucins and of the goblet-cell-derived CLCA1 protein in lungs, which highly suggests that *Pneumocystis* is able to induce lung disease[22–24]. This suggestion has been confirmed in animal models of primary infection where *Pneumocystis* induces a robust immune response and marked pathological changes in the airway, such as mucous cell metaplasia with hypertrophy of epithelial cells and peribronchial and perivascular inflammation and fibrosis, all traits pointing to fungus-induced pulmonary pathology in the immunocompetent host[25, 26]. Moreover, *Pneumocystis* has been related to severity of COPD[27], and associated with asthma[25, 28] and the Sudden Infant Death Syndrome (SIDS)[24, 29]. Nonetheless, the mechanisms involved in the pathological changes during *Pneumocystis* infection have not been fully identified. As a mechanism involved in cellular differentiation in response to lung injuries, Clara cell transdiferentiation in distal airways could be occurring during *Pneumocystis* infection, a possibility that has not yet been studied. Therefore, we aimed to determine the effect of *Pneumocystis* primary infection in Clara cells. Since previous experiments documented increased mucus in response to *Pneumocystis*, we also evaluated the effect of *Pneumocystis* in the Notch pathway, a known regulator of mucin expression.

## Materials and methods

### Ethics

The institutional Animals Welfare Bioethics Committee of the University of Chile, Faculty of Medicine approved the protocol under number CBA #0715. Studies were conducted in accordance with the Animal Protection Law of Chile (Law 20.380) and the Guide for the Care and Use of Laboratory Animals (8th Edition, National Academies Press, Washington DC).

### Animals and experimental design

#### Experiment 1

Thirty five female Sprague Dawley rats of 250 g weight derived from a single colony were obtained from University of Chile, Faculty of Medicine, Animal Research Facility, and subjected to standard housing conditions and HEPA filtered environment (LabProducts Inc, USA), as previously described[26]. Fifteen of them were treated with betamethasone 3 mg/lt for 8 weeks to induce *Pneumocystis* pneumonia (PcP), oxytetracycline 0,625 gr/lt to prevent other infections, and used as Pneumocystis seeder rats. The other 20 rats were timed-pregnant and received tylosin 1 gr/lt until birth of their pups to eradicate other infections. Half of them (Control group or Pc(-)) were additionally treated with trimethoprim-sulfamethoxazole (TMP-SMZ) (80 mg trimethoprim and 400 mg sulfamethoxazole every 5 mL) 15 ml/lt as anti-*Pneumocystis* prophylaxis throughout pregnancy and until sacrifice to secure they remained *Pneumocystis*-free. The other half (*Pneumocystis* group or Pc(+)) were exposed to seeder rats at birth of the colonies, by co-housing. All of the drugs were given in the drinking water. Twelve animals per group were sacrificed under deep anesthesia with ketamine and xylazine on days 40, 60 and 80 of age. Six of them were exsanguinated and their lungs were frozen in RNAlater (Qiagen, USA) until processing. The other six were perfused with 3.7% buffered formalin (pH 7.4), as previously described[26].

#### Experiment 2

Fifteen female Sprague Dawley, 250 grams rats were used as seeding rats, and 30 rats from the same colony were timed-pregnant and exposed to seeding rats, following the same protocol of experiment 1. Pups were divided into four groups: (I) trimethoprim-sulfamethoxazole (TMP-SMZ group), (II) *Pneumocystis* (Pc group), (III) trimethoprim-sulfamethoxazole+valproic acid 300 mg/kg/day in drinking water after weaning (TMS+VPA group) and (IV) *Pneumocystis+*valproic acid (Pc+VPA group). Dose of VPA (Depakene, Abbott) was obtained from a previous assay that used from 100 mg/kg/day (low dose) and 300 mg/kg/day (high dose)[30]. Sacrifice was performed as described in experiment 1, at days 60 and 80 of age (n = 6 in every group).

### *Pneumocystis* detection

All seeder, immunosupressed rats presumed to have *Pneumocystis* pneumonia (PcP) were sacrificed after co-housing and their lungs removed and PcP confirmed by Gomori Grocott Silver Methenamine and Hematoxylin-Eosin stains [31]. For experimental rats with primary infection and control rats, the total genomic DNA was isolated from lung tissue using QIAmp DNA mini kit (QIAGEN, USA). *Pneumocystis carinii* specific primers PAZ102-X and PAZ102-L1R (Table 1) were used for nested polymerase chain reaction (nPCR), including primers for rat actin as internal control[26].

**Table 1:** Primers used for Pneumocystis n-PCR and mucins qPCR.

	Primers	Reference
pAZ102X	5’-GTGAAATACAAATCGGACTAGG-3’	[31]
pAZ102L1R	5’-CTCTCGACTCCTCACCTTAT-3’	[31]
*Muc5ac*	Forward 5’-ACCACGGATATCAGAACCAGC-3’, Reverse 5’-TGTCAAGCCACTTGGTCCAG-3’	This study
*Muc5b*	Forward 5’-CCTGAAGTCTTCCCCAGCAG-3’, Reverse 5’-GCATAGAATTGGCAGCCAGC-3’	This study

### Analyses in lung tissue sections

#### Indirect immunofluorescence (IIF)

Coronal sections of formalin fixed lung at the level of the hilum were made so the lung was divided in two hemilungs. The ventral hemilung was embedded in paraffin and 3 m consecutive histology sections were obtained from the ventral hemilung starting from surface towards the center. The sections were deparaffinized and heated to 95°C in citrate buffer 10 mM, pH 6.0 for 30 minutes for antigen retrieval, and cooled in citrate buffer at 4°C for 10 minutes. 1% bovine serum albumin (BSA; A7888; Sigma-Aldrich) for 30 minutes was used to block nonspecific binding sites. Slices were immunostained using the following primary antibodies: mouse anti-Muc5ac (1:100, ab3649, Abcam, UK), goat anti-Muc5b (1:100, sc135508, Santa Cruz Biotechnology) as markers of Goblet cell phenotype[8, 32], and rabbit anti-CC10 (1:100, ab40873, Abcam, UK) as marker of Clara cell phenotype[2]. Rabbit anti-Ki67 (1:100, PA5-19462, ThermoFisher Scientific, USA) was used as marker of mitosis[33]. Rabbit anti-activated Notch1 (NICD) (1:50, ab52301, Abcam, UK). FITC or rodamine conjugated secondary antibodies were used (Jackson ImmunoResearch Laboratories, Inc., USA). Nuclei were stained with DAPI (1:500, D1306, ThermoFisher Scientific, USA). Brain and stomach were used as positive controls and primary antibodies were omitted to be used as negative controls. Slices were mounted with FluorSave (345789, Calbiochem, Merck Millipore, USA).

#### Alcian Blue-PAS stain

Formalin-fixed paraffin embedded 3 μm thick coronal lung sections were deparaffinized and stained with Alcian Blue/periodic acid (PAS) pH 2.5 (Alcian Blue 8GX, A3157, Sigma-Aldrich and Fucsina Certistain, C.I. 42510, Merck Millipore, USA). An average of 22 (range 12 to 35 sections per rat) small airway images were analyzed.

#### Morphometric analyses

Between 12 and 35 pictures of 50–250 m diameter bronchioles [34] were obtained from a single longitudinal slide section cut parallel and well before reaching the central bronchi. An average of 22 airways per rat were analyzed by one observer who was unaware respect to the *Pneumocystis* status of the microscopy slides. The proportion of rats with epithelial mucins in distal airways and the number of CC10 immunoreactive cells divided by the length (in mm) of basal membrane were determined in an OLYMPUS BX60 epifluorescence microscope using Image-Pro Plus 5.1.2 software. Co-localization images were obtained in confocal microscope (Carl Zeiss, LSM700) using Fiji software (ImageJ 2.0.0-rc-54/1.51h).

### Analyses in fresh frozen lung tissue

#### Western blot

Lung tissue (200 mg) was homogenized in RIPA lysis buffer (100 ml PBS 1x pH 7.2, 1% NP-40, 0.5% sodium deoxycholate, 0.1% SDS, supplemented with protease inhibitors). Total protein was quantified using Bradford assay (Bio-Rad, Hercules, CA). Samples were subjected to SDS-PAGE electrophoresis, using a 15% acrylamide gel for CC10 and 10% acrylamide for Notch1. Seventy five μg of protein were loaded for CC10 and 50 μg for Notch1. They were transferred to PVDF membranes and blocked with 5% nonfat milk. The following primary antibodies were used: mouse anti-Muc5ac (1:2000, sc-21701, Santa Cruz Biotechnology), goat anti-Muc5b (1:2000, sc-135508, Santa Cruz Biotechnology), rabbit anti-CC10 (1:4000, ab40873, Abcam, UK), goat anti-Notch1 (1:2000, sc-6015, Santa Cruz Biotechnology), and Anti-Actin I-19 (1:2000, sc-1616, Santa Cruz Biotechnology, USA). Secondary antibodies conjugated to horseradish peroxidase (Santa Cruz Biotechnology) were used. Brain and stomach were used as positive controls. Water without antibodies was used as negative control. Proteins were detected by Pierce, ECL Western Blotting Substrate (Pierce Biotechnology, Rockford, IL). Images were film-captured. Density of band was measured using Fiji software (ImageJ 2.0.0-rc-54/1.51h) and protein amounts were normalized with the actin signal.

#### Real Time PCR

Total RNA was extracted using RNeasy Mini Kit (Qiagen, USA). Samples were treated with DNase I (ThermoFisher Scientific, USA). 1 μg RNA was reversely transcribed to cDNA using SuperScript. II First-Strand Synthesis System for RT-PCR (Invitrogen, Life Technologies, USA). Muc5ac, Muc5b, CC10, Notch1 or Hes1 genes were amplified by qPCR using Fast SYBR Green Master Mix (Applied Biosystems, USA), in StepOne. Real-Time PCR System (Applied Biosystems, USA). Primers used are specified in Table 1. Brain and stomach were used as positive controls. DEPC-treated water without primers was used as negative control. Gene expression was calculated using the 2^-ΔΔCT^ method and values were normalized by actin. Reactions were performed in duplicate.

### Statistical analysis

Distribution of data was determined with Shapiro-Wilk test. ANOVA was used for parametric distribution, with Bonferroni’s multiple comparisons test. Kruskal-Wallis was used for nonparametric distribution, with Dunn’s multiple comparisons test. *p*<0.05 was considered statistically significant. Analyses were performed using Prism GraphPad 7.0 Software (GraphPad Software Inc, San Diego, CA, USA).

## Results

### *Pneumocystis carinii* associated to increased expression of epithelial mucins in the distal airway

**Fig 1:**
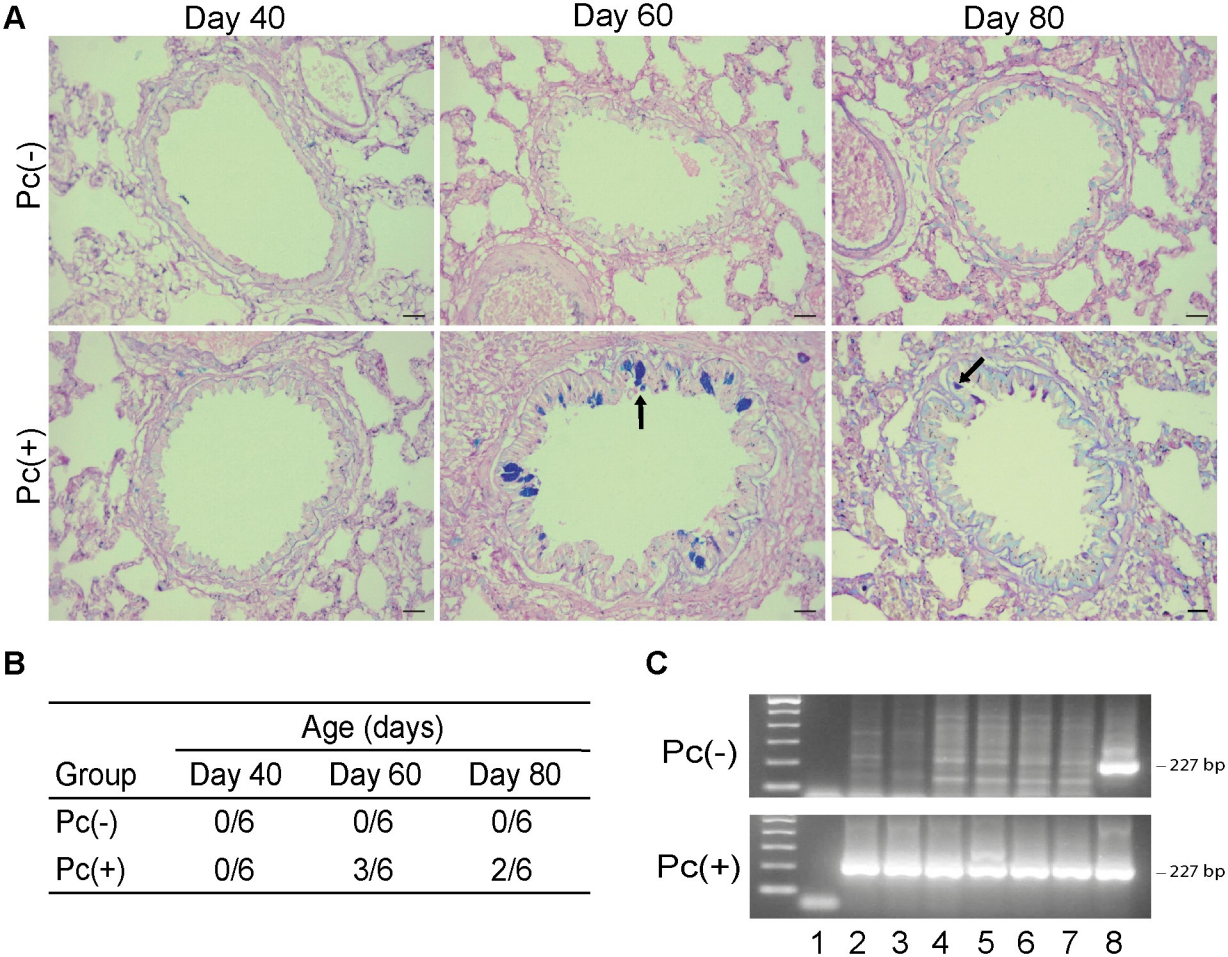
Mucins in distal airway of rats and *Pneumocystis* DNA in infected rats. (**A**) Mucins as identified by Alcian Blue/PAS stain in distal airways in Pc(-) control rats and Pc(+) *Pneumocystis*-infected rats, at different days of infection. Representative images. Epithelial mucins in the day 60 of infection are indicated by the arrow. Scale bars = 10 μm. Magnification 20x. (**B**) Number of rats with epithelial mucins in distal airway related to total number of rats. n = 6. **C**. Presence of DNA of *Pneumocystis* determined by nPCR in Pc(-) and Pc(+) rats, at different times of infection. n = 6. A representative experiment of days 60 and 80 is shown. Lane 1 = negative control, lane 2 = day 60 sample 1, lane 3 = day 60 sample 2, lane 4 = day 60 sample 3, lane 5 = day 80 sample 1, lane 6 = day 80 sample 2, lane 7 = day 80 sample 3, lane 8 = positive control.

In order to confirm *Pneumocystis* primary infection in the group of exposed rats, presence of *Pneumocystis carinii* was determined by n-PCR. DNA of *Pneumocystis* was found in 100% of Pc(+) rats, whereas none of the negative control Pc(-) rats were positive (Fig 1C).

**Fig 2:**
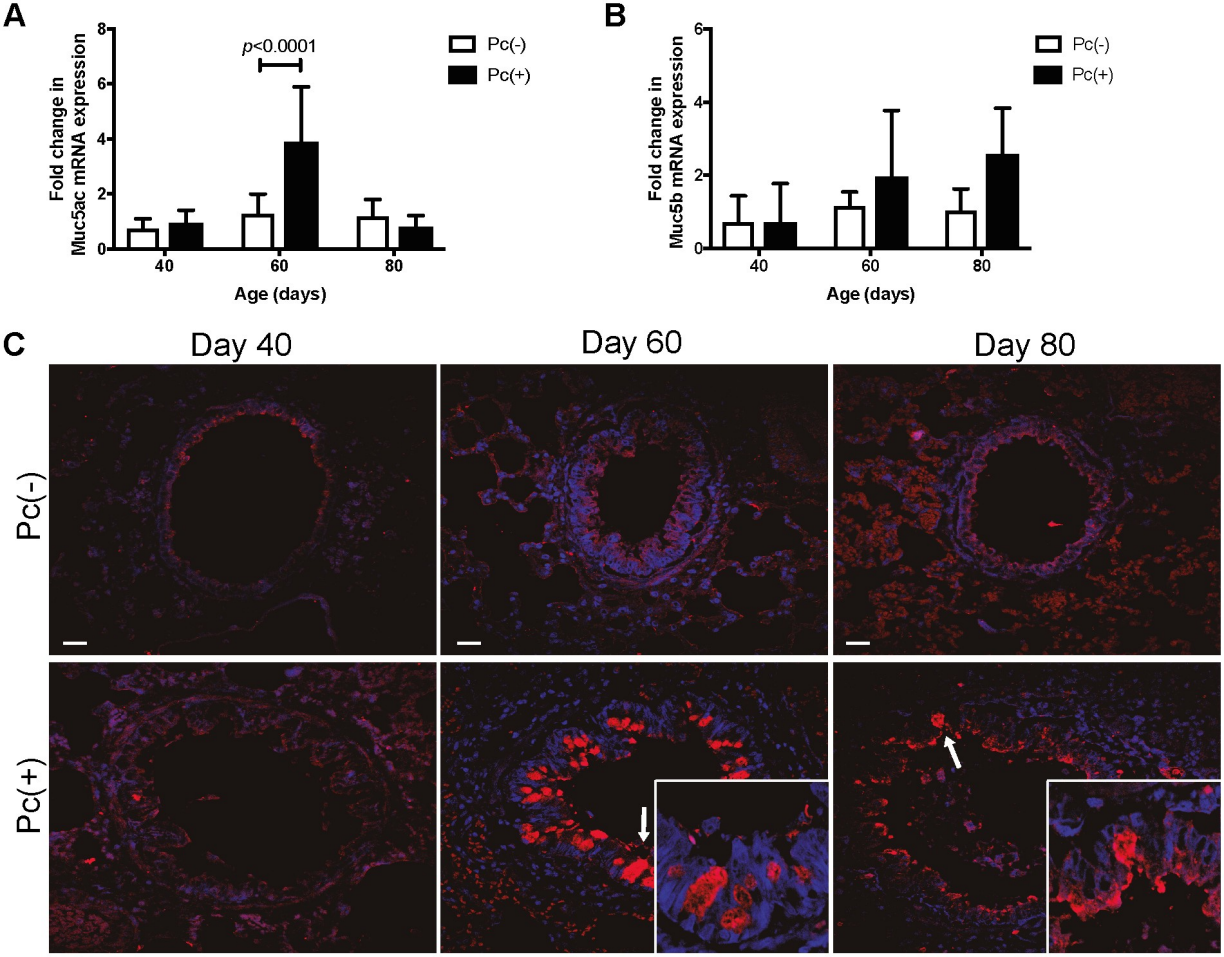
Messenger RNA levels of *Muc5ac* and *Muc5b* in whole lung homogenates of rats and *Muc5b* in distal airways of rats. The levels of expression of the Mu5ac and Muc5b mucins in whole lungs of Pc(-) and Pc(+) rats was assessed by qPCR and the presence of Muc5b in distal airway was determined in Pc(-) and Pc(+) rats, at different days of infection by IIF with antibody anti-Muc5b and nuclear stain with DAPI. (**A**) mRNA levels of *Muc5ac*. Data are shown as mean ± standard deviation (SD). (**B**) mRNA levels of *Muc5b*. Data are shown as median and interquartile range. For all measurements n = 6. (**C**) Representative images. Muc5b immunoreactive cells in the days 60 and 80 are shown (arrows and insets). Scale bars = 10 μm. Magnification 20x, insets 100x.

The proportion of rats per group with detectable epithelial mucins in distal airway was determined. An increase in this proportion was observed in the *Pneumocystis* infected group, with the highest rate in the day 60 of infection (Fig 1A and 1B). Next, we determined mRNA of *Muc5ac* and *Muc5b* by RT-PCR. A 2.6 fold increase in mRNA levels of *Muc5ac* that was statistically significant (*p*<0.0001) when compared to the Pc(-) group was observed in day 60 of infection (Fig 2A). A non-statistically-significant increase in mRNA levels of *Muc5b* was observed in days 60 and 80 of infection (Fig 2B). In addition, Muc5b immunoreactive cells were detected by IIF. Muc5b positive cells were detected at days 60 and 80 of infection in distal airway, with a higher prevalescence at day 60 (Fig 2C). Muc5ac detection was attempted without success due to excessive IIF background signal.

### *Pneumocystis carinii* is associated to reduction in CC10 levels, with no changes in frequency of Clara cells.

**Fig 3:**
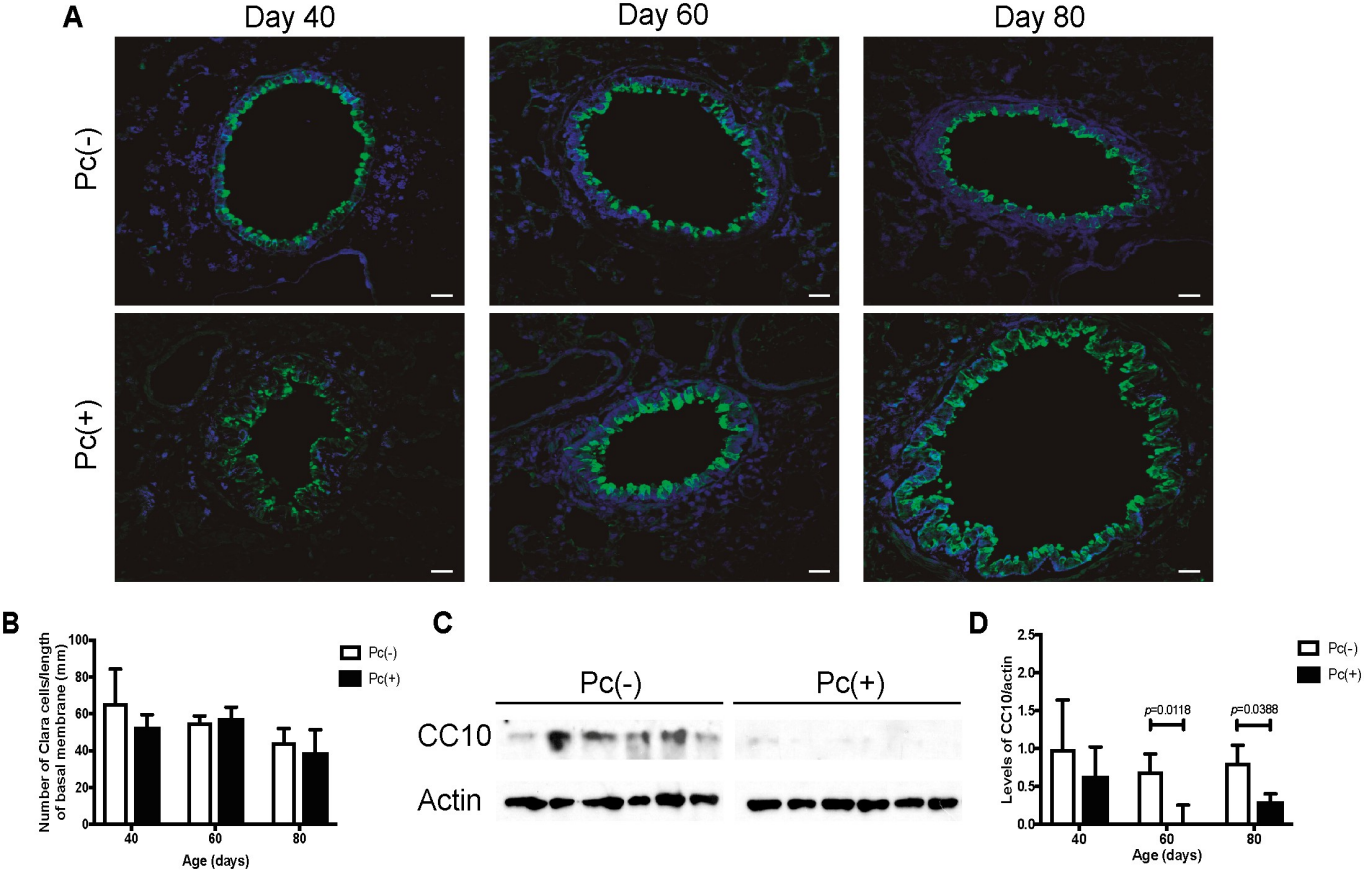
Clara cells in distal airway and protein levels of CC10 in lungs of rats. Presence of Clara cells in distal airway was determined in Pc(-) and Pc(+) rats, at different days of infection by IIF with antibody anti-CC10, nuclear stain with DAPI. Protein levels of CC10 were determined in whole lungs of Pc(-) and Pc(+) by western blot. (**A**) Representative images of Clara cells. Scale bars = 10 μm. Magnification 20x. (**B**) Frequency of Clara cells assessed by quantification of cells by length of basal membrane (mm). Data are shown as mean ± SD. (**C**) Representative images of CC10 levels in day 80 of infection. (**D**) Protein levels of CC10. Data are shown as median and interquartile range. For all measurements, n = 6.

The presence of mucins and increase of expression of mucin genes in the distal airway in infected animals shown in the previous experiment could be the result of an increase in goblet cells at the expense of Clara cells in the epithelium. In order to explore this possibility, the frequency of Clara cells in distal airway was determined by measurement of number of CC10 immunoreactive cells divided by length of basal membrane. No change in frequency of Clara cells was found between Pc(+) and negative control group (Fig 3A and 3B). Then, CC10 protein levels were determined by western blot. Strikingly, in spite that no reduction of Clara cells occurred, CC10 protein in distal airway was significantly reduced in day 60 (*p* = 0.0118) and 80 of infection (*p* = 0.0388) when compared to the Pc(-) group (Fig 3C and 3D).

### The increase in distal airway mucins during *Pneumocystis* infection is related to colocalization of CC10 with Muc5b and independent of mitosis, which suggests cellular transdifferentiation process.

**Fig 4:**
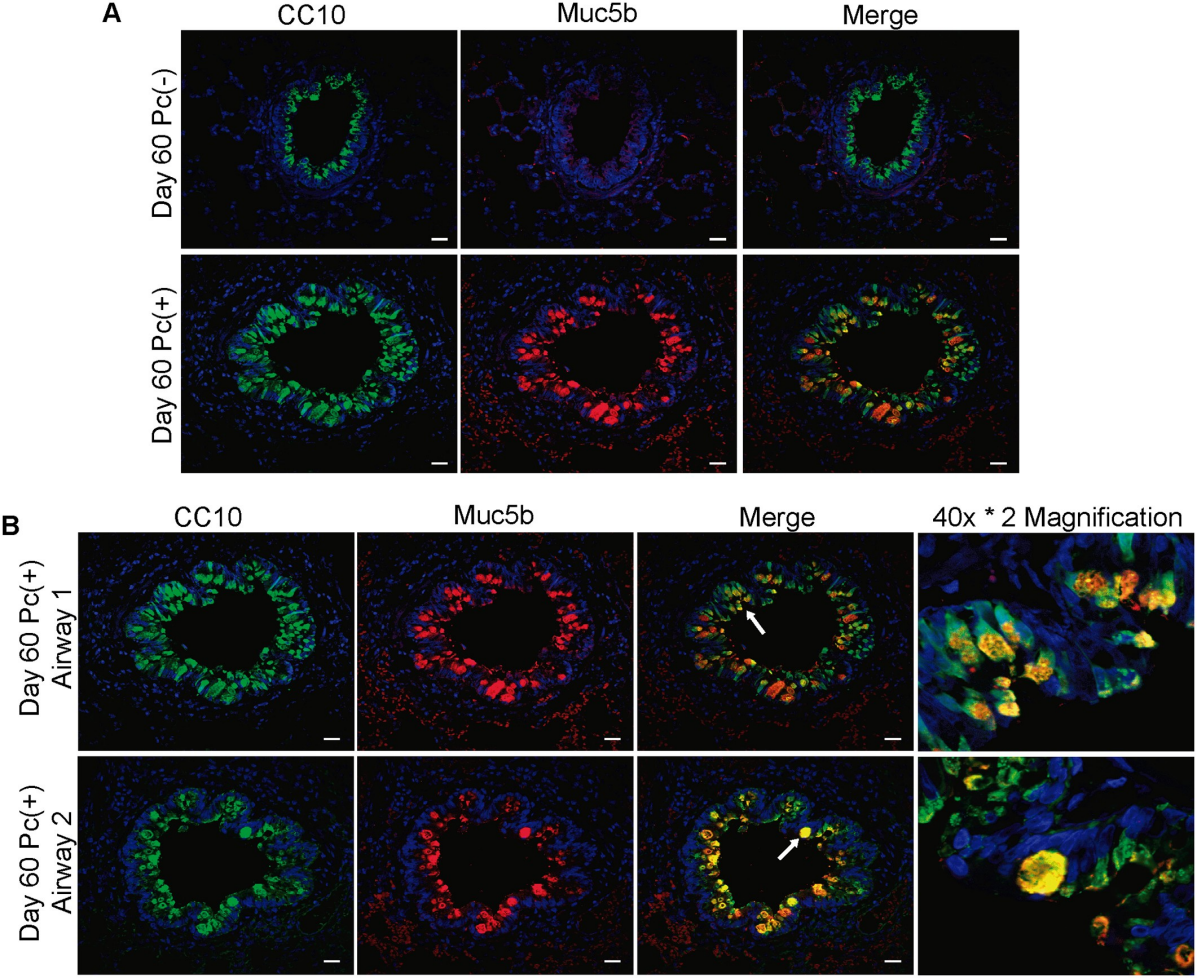
Colocalization of CC10 and Muc5b in distal airway of rats. Colocalization of CC10 and Muc5b in distal airway was determined in Pc(-) and Pc(+) rats, at different days of infection by IIF with antibodies anti-CC10 and anti-Muc5b, nuclear stain with DAPI. (**A**) Representative images of Pc(-) and Pc(+) groups. Scale bars = 10 μm. Magnification 20x. (**B**) Representative images of day 60 of infection. Two distal airways immunoreactive to CC10 and Muc5b are shown. Arrows in merge indicate immunoreactive cells for both markers, magnified to 40x*2 at the right side.

**Fig 5:**
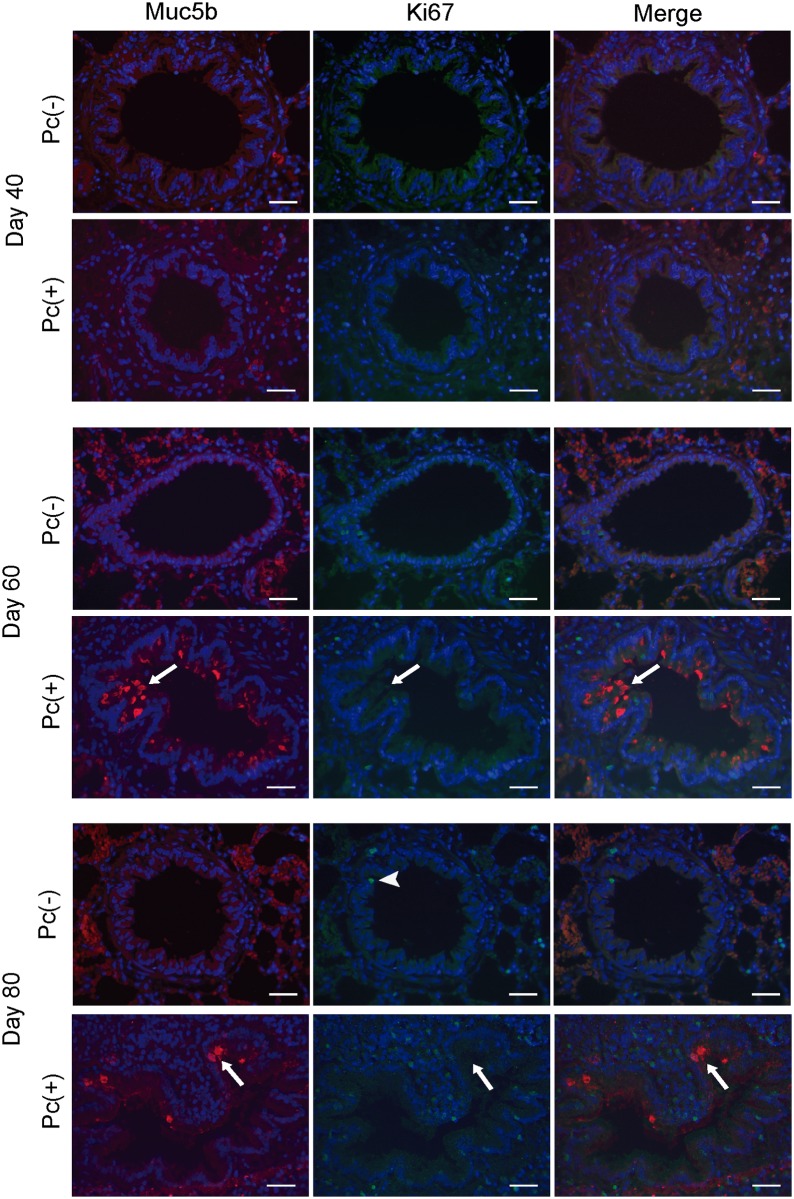
Muc5b and Ki67 in distal airway of rats. Colocalization of Muc5b and Ki67 in distal airway was determined in Pc(-) and Pc(+) rats, at different days of infection by IIF with antibodies anti-Muc5b and anti-Ki67, nuclear stain with DAPI. Representatives images of Pc(-) and Pc(+) groups at days 40, 60 and 80 of infection are shown. Scale bars = 25 μm. Magnification 40x. Arrows indicate immunoreactive cells to Muc5b but not to Ki67. Arrowhead indicates an immunoreactive cell to Ki67 but not to Muc5b.

In other models, co-expression of Clara cell and goblet cell markers has been found as indicator of an ongoing transdifferentiation process. Thus, CC10 and Muc5b immunoreactive cells were simultaneously determined by IIF in epithelia. CC10 and Muc5b positive cells were detected mainly in *Pneumocystis* infected epithelial cells at the day 60 of infection (Fig 4A). Cells that were positive for both markers were observed documenting co-localization of CC10 and Muc5b markers (Fig 4B). Alternatively to transdifferentiaton from Clara cells, goblet cells could be also originated by cellular proliferation. Thus, we also evaluated mitosis in Muc5b immunoreactive cells by IIF using antibodies against the mitosis marker Ki67 and anti-Muc5b. No Ki67 and Muc5b immunoreactive cells were found in distal airways at any day of infection (Fig 5).

### *Pneumocystis carinii* infection is not associated to suppression of Notch1 pathway in distal airways

**Fig 6:**
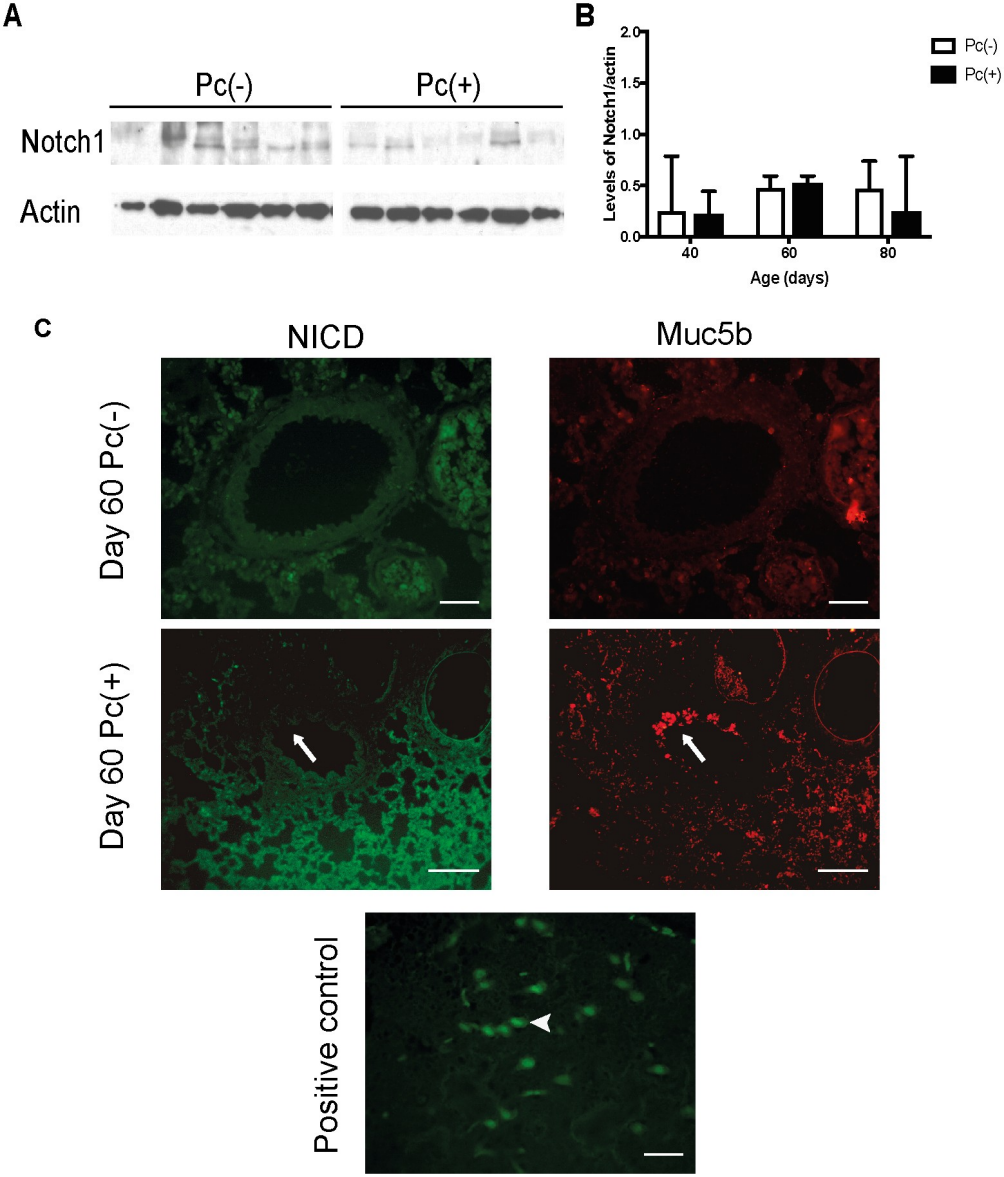
Components of Notch pathway in the whole lung and distal airway. Protein levels of Notch1 were determined in the whole lungs of Pc(-) and Pc(+) groups, at different days of infection by western blot. Localization of activated Notch intracellular domain (NICD) was determined in distal airway by IIF with antibodies anti-activated Notch1 (NICD) and anti-Muc5b. (**A**) Representative experiment of protein levels of Notch1 in the day 40 of infection. (**B**) Densitometric analysis of the Notch1 western blots. Data are shown as median and interquartile range. (**C**) Representatives images of NICD and Muc5b of Pc(-) and Pc(+) groups at day 60 of infection, and positive control (brain). Scale bars = 25 μm and 100 μm, magnification 40x and 10x, respectively. Arrows indicate immunoreactive cells to Muc5b but not to NICD. Arrowhead in positive control (brain tissue) shows an immunoreactive cell to NICD. For all experiments, n = 6.

We assessed whether the induction of mucus observed in infected rats is the result of downregulation of Notch1. The protein levels of Notch1 as determined by western blot detected no differences between Pc(+) and Pc(-) groups at any day of infection (Fig 6A and 6B). We also determined translocation of the Noch activated form NICD to the nucleus by localization of NICD in cells of distal airways using IIF with antibodies anti-NICD and anti-Muc5b. NICD immunoreactive epithelial cells were not frequent in the airways, and the NICD mark was cytoplasmic, without differences between Pc(-) and Pc(+) groups.

### Valproic acid (VPA) increased levels of epithelial mucins in distal airways in few *Pneumocystis* infected rats

**Fig 7:**
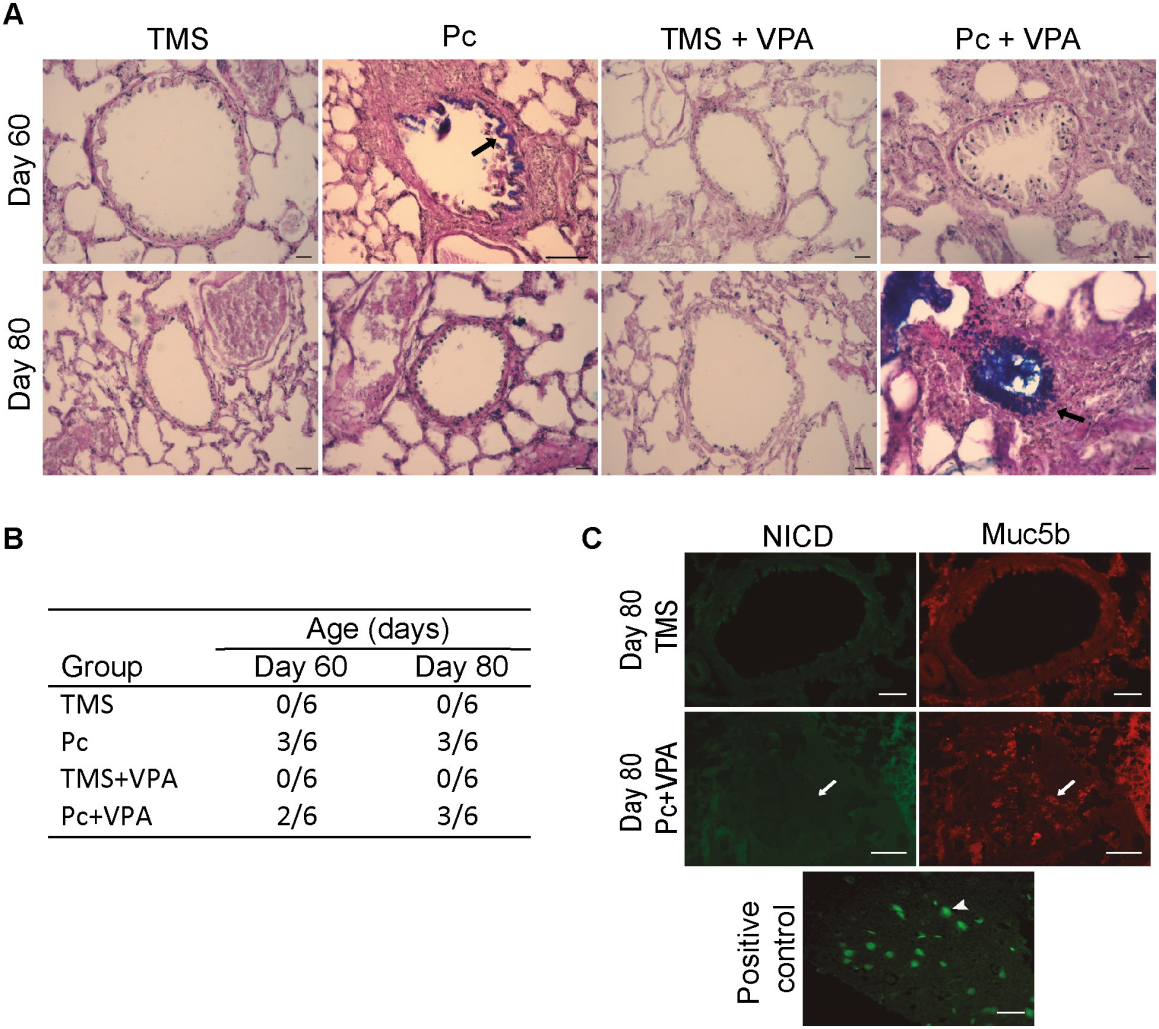
Mucus and NICD in distal airway of rats treated with valproic acid. Mucins in distal airway were determined in TMS, Pc, TMS+VPA and Pc+VPA groups, at days 60 and 80 of infection by Alcian Blue/PAS stain. VP groups received high VPA dose (300 mg/kg/day). NICD was determined by IIF with antibodies anti-activated Notch1 (NICD) and anti-MUC5B. (**A**) Representative images. Epithelial mucins in the days 60 of Pc group and 80 of Pc+VPA group are shown (black arrows). Scale bars = 100 μm and 10 μm, magnification 10x and 20x, respectively. (**B**) Proportion of rats with epithelial mucins in distal airways. (**C**) Representatives images of TMS (control), Pc+VPA groups at day 80 of infection and positive control (brain). Scale bars = 25 μm and 100 μm, magnification 40x and 10x, respectively. Arrows indicate immunoreactive cells to Muc5b but not to NICD. Arrowhead in positive control shows an immunoreactive cell to NICD. For all experiments, n = 6.

Valproic acid (VPA) is an inducer of Notch signaling by inhibition of the Histone deacetylase [35, 36]. To confirm the Notch independence of the *Pneumocystis* effects on distal airway cells we evaluated the effect of VPA on *Pneumocystis*-induced mucin production. A mild increase in the proportion of rats with epithelial mucins was observed in the *Pneumocystis*-infected rats at day 60. The number of rats positive for Alcian Blue staining was similar (3/6) in the Pc and in the Pc+VPA groups on day 60 and the same is true for day 80. Nonetheless, although the frequency of rats staining for mucins was similar, VPA stimulation resulted in a high increase in mucin production with detection of luminal mucus mark in some animals at day 80 (Fig 7A and 7B). Translocation of activated Notch1 (NICD) to the nucleus was not observed in distal airway goblet cells. Scarce NICD mark was detected in the cytoplasm of epithelial cells, with no differences between groups. NICD was not observed in the cytoplasm nor in the nucleus of MUC5B immunoreactive cells (Fig 7C).

## Discussion

The first part of this work documented an increase in the proportion of rats with epithelial mucins in distal airway observed in the day 60 and 80 of infection, before the point of highest burden of *Pneumocystis* occurring after 80 days in a previous model of primary *Pneumocystis* infection [26]. The present results are similar to previous studies in showing increase in epithelial mucins in the day 60 of infection by *Pneumocystis*[26]. Of note, distinct niches of murine airway epithelial cells may express different mucus markers [37] and may also explain differences in mucin expression between rat studies that study different sections of the lung [37]. This study focuses in distal airways with diameter 50–250 μm and previous studies showed mucins or mucus in total airways [25, 26, 28]. Pathological changes in distal airways are relevant to study because obstruction of this region affects the distribution of ventilation and may lead to small airway closure [38]. Methacholine sensitivity has been explored in immunocompetent mice sensitized with OVA and infected with *Pneumocystis* via the intratracheal route and document that this fungus impressively increases airway sensitivity to methacholine [25, 39]. This increase is comparable to the effect of the common allergen House Dust Mite [25]. Whether this effect occurs in rats spontaneously infected with *Pneumocystis* or other mammals in addition to mice has not been determined. The level of epithelial mucins observed in this study was lower to that of previous studies by our group [26], and this difference can be attributed to variations in the timing of greater mucin induction. This timing may vary between models of primary infection that use the aerial route of natural contagion without a fixed inoculum as occurs with *Pneumocystis* infection in infants.

The increase in *Muc5ac* mRNA levels and the documentation of cells coexpressing CC10 and Muc5b observed in small airways in this model is relevant because distal airways lack mucus expression driven by goblet cells, as such cells are normally absent in distal airways [2, 12]. Furthermore, there is increasing evidence that chronic airway disease may be influenced by early life events [40]. This study documented mucus expression characterized by colocalization of CC10 with Muc5b in distal airway epithelium, mostly in day 60. Reduction of the CC10 protein in the whole lungs in the days 60 and 80 of infection, along with no changes in frequency of Clara cells in the distal airway suggest that CC10 and Muc5b were expressed in the same cell, i.e. Clara cell. Clara cells have previously reported to produce mucins in models of obstructive lung diseases. Mucin expression in response to challenge in allergic mice models by Clara cells, with a high presence of the mucin Muc5ac has been described [2, 41]. In the allergic rhinitis murine model occurs a transdifferentiation of Clara cell with a concomitant expression of the trefoil factor family peptide 1 (TFF1) goblet cell marker in some airway regions. TFF1 works together with mucins for mucus formation. In this model, goblet cells with intermediate phenotypes coexpressing CC10 and TFF1 in response to allergen are observed [42]. Specifically, coexpression of CC10 and Muc5b as observed here has been reported before. Basal colocalization of CCSP and MUC5B in distal airway of healthy human lungs was recently reported [43]. This basal level of Muc5b expression was not detected in our control group possibly because rats in this experiment were breathing HEPA-filtered air and not exposed to environmental contaminants as may have been the case of humans [43]. Increases in CSSP and Muc5b co-expressing cells occurrence and/or overexpression of Muc5b in such cells have been documented in response to mutations in *Cftr* and *Munc13-2* in murine models [44, 45]. Thus, it seems that induction of Clara cell expression of mucins may also occur as a result of an infectious setting or general immunological impairments [2, 7].

The marker of proliferation Ki67 was not observed in goblet cells from distal airways in the present study, suggesting that the increase epithelial mucins in the distal airway was independent of mitosis. Ki67 is specific of mitosis and is not found in G0 cell division phase [33]. This has been previously demonstrated in model of asthma induced by ovalbumin [2]. The absence of mitosis supports transdifferentiation from Clara cells as a mechanism involved in the increase of epithelial mucins during *Pneumocystis* infection [8]. The process of transdifferentiation from Clara to goblet cells has been reported to be mediated by Notch1. Specifically, suppression of Notch1 pathway induces differentiation to goblet cells and mucin expression [12, 16]. However, no changes in expression of Notch1, nor nuclear translocation of NICD between groups, and NICD was not observed in Muc5b immunoreactive cells in the present study. Moreover, signal of activated Notch1 was sparse in epithelium of distal airway. These findings suggest that *Pneumocystis* infection is not associated to suppression of Notch1 pathway in epithelium of distal airway. To corroborate these results, an inducer of Notch pathway, VPA, was used. Contrary to what would have been expected if the Notch pathway were involved, a robust increase in the levels of epithelial mucins was observed in distal airways, and this boosting required the presence of *Pneumocystis*, because the uninfected group treated with VPA did not exhibits this increase in mucins. The high increase in mucus observed in some *Pneumocystis* infected animals that received VPA was not anticipated and suggests other mechanisms might be involved. Epigenetic functions have been described for VPA[46]. VPA effects on Histone deacetylase [35, 36] may likely change dramatically nuclear DNA topology leading to pleiotropic effects on gene regulation. Certainly, the underlying molecular mechanisms involved in VPA induction of Notch1 signaling have not been fully defined [47].

We were unable to document changes in Notch activation related to *Pneumocystis* infection in this study and results therefore show that *Pneumocystis* associated mucus increase was not associated to changes in Notch1 pathway in the epithelium of distal airway, suggesting that the inferred transdifferentiation in Clara cell function associated to *Pneumocystis* was independent of Notch pathway downregulation function. The use of VPA to activate the Notch pathway is widely stablished in different cellular types, such as Clara cell and carcinoma models. Although the effect of this molecule is highly cell specific, a clear role of VPA in activating the Notch pathway and blocking Clara cell transdifferentiation has been reported [10]. In addition, we also determined the transnucleation of the Notch intracellular domain both in *Pneumocystis* infected and noninfected animals. Results showed no evidence of Notch translocation in either group, suggesting that in this model the Notch pathway has a low level of basal activation per se. This also supports the independence of Notch in *Pneumocystis*-stimulated mucus production. Nonetheless, the mechanism by which VPA activates Notch is largely unclear [48]. There has been proposed that VPA may activate the Notch pathway at several levels, including antirrepresion of the Hes-1 promoter and direct promotion of Notch translocation [48]. Moreover, VPA is a molecule with pleiotropic cellular effects, as reported in high throughput studies that show activation and repression of many pathways in response to VPA exposure [49]. Thus, although in our model VPA should be activating Notch, is not clear which pathways may be responsible for the outcome observed, which drive the mucus overexpression in VPA treated animals. Our findings suggest that the *Pneumocystis* effect on mucus induction is achieved through the stimulation of a different signaling pathway. Direct airway stimulation with IL13 induces selective expression of STAT6 and mucus production in Clara cells [50]. Swain et al. demonstrated that STAT6 stimulation by *Pneumocystis* is a potent inductor of mucus innate responses [28], and in agreement with this, us and others have shown that the Il13 STAT6 CLCA1 pathway activation is associated to *Pneumocystis* in human tissue and rat models of primary infection [22, 24, 25]. Hence, is clear that Notch-independent mechanisms can mediate mucin induction [10, 51]. Regarding this, we have recently documented that kaempferol, a STAT6 pathway inhibitor, was associated to partial reduction of levels of mucins during *Pneumocystis* infection [23]. Nonetheless, whether STAT6 or other pathways are specifically involved in Clara cell expression of mucins described here remain to be elucidated.

In summary, our findings document the involvement of Clara cells in mucus secretion during primary infection by *Pneumocystis* and therefore contribute to the understanding of cellular and molecular mechanisms involved in the pathogenic traits developed. *Pneumocystis* may induce mucus via a Notch-independent mechanism triggering transdifferentiation of Clara cells. Further characterization of the host response resulting in expression of mucins during *Pneumocystis* infection is needed.

## Supporting information

S1 Dataset Quantitative values for Figs 2, 3, and 6. (XLSX)
